# Help Father

**Published:** 1866-12

**Authors:** 


### HELP FATHER.

"I do not mind being sick so much, nor the pain connected with it, but father is getting old, and he needs my help at the store."

This was the sentiment of a young man the other day, who was suffering a temporary disablement. There was something beautiful in it.

Every night about dark, a small boy with a light ladder and a box of matches, may be seen running along the street. He stops at each lamp post, lights the gas, and then runs to another; he does not loiter; does not stop to talk with other boys, or to gaze at the splendid equipages which are yet returning from the Park along the Avenue. He is the son of the man whose business it is to light the city lamps. No hired boy could possibly be procured who would perform the duty with such alacrity, and fidelity, too; for not long ago he passed a lamp-post a rod or two and on looking back, he discovered it was burning too high. He at once ran back and adjusted it properly. What if it did burn too high; it was nothing to him, nor to the city, nor to his father; but suppose one of the Directors of the gas companies had chanced to see that wasting light, his father might have lost his place. A lesson may be learned here which would add a million fold to family happiness, and it is this—it increases the affections of families to have the sons and daughters brought up to assist their parents in household duties, or in the means of family support; there grows up a community of interest, each feels that he is helping the others, and the affections naturally go out towards those who help us. If a mother has no other use of her daughter, she should require her to assist in all domestic matters; and sons should be early learned to proffer their services to their parents, even if it be to save a few steps in walking, or to make the accomplishment of any work in hand less laborious; in short, children should be so educated that it shall be to them a duty, a pleasure and a happiness to help their parents, and to do it with alacrity, with a spontaneous promptitude which never gives time to be asked. But most parents will plead guilty to the charge, that instead of requiring their children to help them, and wait upon them in many little ways, they too often prefer to wait on or help themselves.

There is a feeling, which if expressed in words would read thus—"The child may be engaged in something else of its own, or may not like to do it, and I can almost as easily do it myself; at all events I won't divert him from his own objects." Many a mother has made a bed in weariness, or cooked a dinner, or swept a room, or washed a garment, or set up half the night in sewing, patching or knitting, when her daughters could have shared the labor and made it easy to both; but that daughter's dress might have been soiled, or she had a visitor, or was going to take a walk, or was finishing a story.—Mothers, remember this one important truth—the most self-sacrificing parents are oftenest brought to an old age of grief by the misconduct of both sons and daughters.

Selfish children, those who are not allowed to bear the famly burdens soon begin to feel that it is not their place and that their parents ought to devote themselves to their comfort and happiness, and the sentiment expresses itself in words sometimes, "It is no more than they ought to do." "Other children's parents do it, and why not mine?" Nothing is more true than that those children who take the least interest in household matters, or in the business of the family, are the most selfish, the most ungrateful, and the least unlikely to grow up useful members of society, while those who are always ready to help their parents, to share their labors. will grow up more and more affectionate, more and more worthy of parental love, and are very certain in after life to be found the praise of the community in which they live.

